# Resveratrol Inhibited Hydroquinone-Induced Cytotoxicity in Mouse Primary Hepatocytes 

**DOI:** 10.3390/ijerph9093354

**Published:** 2012-09-19

**Authors:** Da-Hong Wang, Yoshie Ootsuki, Hirofumi Fujita, Masahiro Miyazaki, Qinxia Yie, Ken Tsutsui, Kuniaki Sano, Noriyoshi Masuoka, Keiki Ogino

**Affiliations:** 1 Department of Public Health, Okayama University Graduate School of Medicine, Dentistry, and Pharmaceutical Sciences, 2-5-1 Shikata-cho, Okayama 700-8558, Japan; Email: gms422014@s.okayama-u.ac.jp (Y.O.); qinxia8402@yahoo.co.jp (Q.Y.); kogino@md.okayama-u.ac.jp (K.O.); 2 Department of Cytology and Histology, Okayama University Graduate School of Medicine, Dentistry and Pharmaceutical Sciences, 2-5-1 Shikata-cho, Okayama 700-8558, Japan; Email: fujita00@md.okayama-u.ac.jp; 3 Department of Food and Nutrition, Faculty of Human Services, Okayama Gakuin University, 787 Aruki, Kurashiki 710-8511, Japan; Email: mmhkrsyz@owc.ac.jp; 4 Department of Genome Dynamics, Okayama University Graduate School of Medicine, Dentistry, and Pharmaceutical Sciences, 2-5-1 Shikata-cho, Okayama 700-8558, Japan; Email: tsukken@cc.okayama-u.ac.jp; 5 Department of Neurogenomics, Okayama University Graduate School of Medicine, Dentistry, and Pharmaceutical Sciences, 2-5-1 Shikata-cho, Okayama 700-8558, Japan; Email: kuniaki@md.okayama-u.ac.jp; 6 Department of Life Science, Okayama University of Science, 1-1 Ridai-cho, Okayama 700-0005, Japan; Email: masuokan@dls.ous.ac.jp

**Keywords:** resveratrol, hydroquinone, cytotoxicity prevention, CYP 2E1, primary culture of mouse hepatocytes

## Abstract

Hydroquinone (1,4-benzenediol) has been widely used in clinical situations and the cosmetic industry because of its depigmenting effects. Most skin-lightening hydroquinone creams contain 4%–5% hydroquinone. We have investigated the role of resveratrol in prevention of hydroquinone induced cytotoxicity in mouse primary hepatocytes. We found that 400 µM hydroquinone exposure alone induced apoptosis of the cells and also resulted in a significant drop of cell viability compared with the control, and pretreatment of resveratrol to a final concentration of 0.5 mM 1 h before hydroquinone exposure did not show a significant improvement in the survival rate of the hepatocytes, however, relatively higher concentrations of resveratrol (≥1 mM) inhibited apoptosis of the mouse primary hepatocytes and increased cell viability in a dose-dependent manner, and in particular the survival rate of the hepatocytes was recovered from 28% to near 100% by 5 mM resveratrol. Interestingly, pretreatment with resveratrol for longer time (24 h), even in very low concentrations (50 µM, 100 µM), blocked the damage of hydroquinone to the cells. We also observed that resveratrol pretreatment suppressed hydroquinone-induced expression of cytochrome P450 2E1 mRNA dose-dependently. The present study suggests that resveratrol protected the cells against hydroquinone-induced toxicity through its antioxidant function and possibly suppressive effect on the expression of cytochrome P450 2E1.

## 1. Introduction

**Figure 1:**
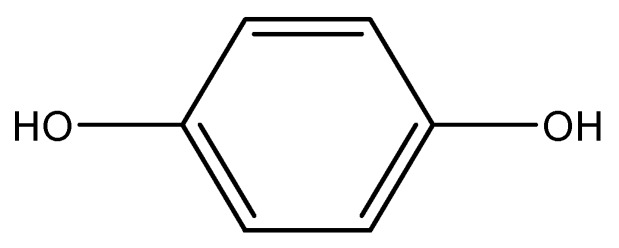
Chemical structure of hydroquinone.

Hydroquinone (1,4-benzenediol, Figure 1), a benzene-derived metabolite, has been widely used in clinical situations and the cosmetics industry because of its depigmenting effect through its influence upon melanocyte metabolism [1,2]. Most skin-lightening hydroquinone creams contain 4%–5% hydroquinone [3]. The U.S. Food and Drug Administration (FDA) has reported that even low concentrations (1%–2%) of hydroquinone can cause exogenous ochronosis [4] and has proposed a ban on over-the-counter (OCT) hydroquinone. The European Committee has banned the use of hydroquinone in cosmetics in 2001 and only physicians and dermatologists can prescribe the hydroquinone formulations (24th Directive 2000/6/EC). Previous studies have demonstrated DNA damage induced by hydroquinone via generation of reactive oxygen species (ROS) [5,6,7,8,9], and hydroquinone-induced carcinogenicity was observed in both mice and rats [10]. 

**Figure 2:**
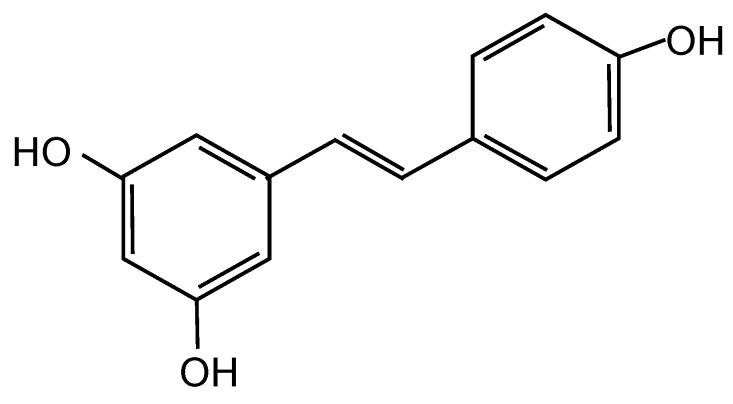
Chemical structure of resveratrol.

Resveratrol (*trans*-3,5′,4-trihydroxystilbene, Figure 2) is one of the naturally occurring polyphenols found in the skins of red grapes and some plants, and it is capable of promoting the activities of many antioxidant enzymes and inhibiting free radical-mediated cellular processes [11]. The potential role of resveratrol in cancer prevention [12] and antiaging activity in *Drosophila melanogaster* and *Caenorhabditis elegans* [13] have been noticed. Leone *et al*. reported that the anticancer effect of resveratrol is exerted by acting as a topoisomerase II poison in proliferating cells to induce DNA double-strand breaks [14]. The present study used mouse primary hepatocytes to examine the effect of resveratrol on hydroquinone-induced cytotoxicity.

## 2. Methods

### 2.1. Chemicals

Resveratrol, hydroquinone, kanamycin sulfate, insulin, dexamethasone sodium phosphate, ethylene glycol tetraacetic acid (EGTA), collagenase, and kanamycin sulfate, were purchased from Wako Pure Chemical Industries (Osaka, Japan). The cell counting kit (WST-8) was from Dojindo Company (Osaka, Japan). All other chemicals were obtained from Sigma Chemical Co. (St. Louis, MO, USA) unless otherwise mentioned.

### 2.2. Mouse Hepatocyte Isolation and Primary Culture

Adult male mice (C3H/AnL) were used in this study. They were anaesthetized intraperitoneally with 9 μL/g body weight of pentobarbital sodium (10% in PBS) and then the liver was first perfused at 37 °C with EGTA at a flow rate of 5.5 mL/minute for 10 minutes and thereafter with collagenase at the same flow rate for 15 minutes as described previously [15]. The yield of isolated hepatocytes was determined with a hemocytometer, and their viability was evaluated with the standard trypan blue exclusion method. The viability of the isolated hepatocytes was around 90%, The isolated hepatocytes (5 × 10^6^) were seeded into 100-mm-diameter plates or 96-well plates containing serum free hepatocyte growth medium (HGM) (Block 1996, Miyazaki 2002) supplemented with 1 μM insulin, 1 μM dexamethasone sodium phosphate, 0.1 mg/mL kanamycin sulfate, and allowed to attach for 24 h before used in the experiments.

### 2.3. Cell Viability Assay

Primary hepatocytes seeded into 96-well plates at a density of 1.2 × 10^4^ per well were used to evaluate the cytotoxic effect of hydroquinone and the preventive effect of resveratrol. Hydroquinone was added to the cells to final concentrations of 0.4 mM and incubated at 37 °C for 2 h. Resveratrol (final concentrations 0.5 mM, 1 mM, 5 mM, respectively) was added to the cells 1h before the addition of hydroquinone and was contained throughout the experiments. Cell viability was determined using the WST-8 assay, based on the reduction of the tetrazolium salt to a water-soluble formazan product by the cellular dehydrogenase [16]. Absorbance was measured at 450 nm (VERSAmax microplate reader). The viability was expressed as a percentage of cell survival in the negative control group based upon the following formula: Survival (%) = (Absorbance of sample – Absorbance of blank)/(Absorbance of negative control – Absorbance of blank). At least three tests were performed in each experiment.

### 2.4. Measurement of Hydroquinone-Induced Apoptosis

Primary hepatocytes seeded into 6-well plates at a density of 5.6 × 10^5 ^per well was exposed to final concentrations from 0.1 mM to 0.4 mM of hydroquinone for 48 h on triplicate plates relative to control, then the cells were stained with Hoechst 33342 at 5 µM of final concentration (Sigma Chemical Co., St. Louis, MO, USA) for 10 min and observed under the fluorescence microscope [17]. The chromatin condensation was expressed as a percentage of the total nuclei in the same field. 

### 2.5. Reverse Transcriptase Polymerase Chain Reaction (RT-PCR)

Total RNA of primary hepatocytes was extracted with ISOGEN (Nippon Gene, Tokyo, Japan). RT-PCR was performed using TaKaRa RNA PCR kit AMV ver. 3.0 with oligo-dT primers according to the manufacturer’s instructions. Primers used for RT-PCR analysis of cytochrome P450 2E1 (CYP2E1) were 5′-TCCCTAAGTATCCTCCGTGA-3′ and 5′-GTAATCGAAGCGTTTGTTGA-3′. PCR amplification was performed for 35 cycles (30 s at 93.5 °C for denaturation, 30 s at 55 °C for annealing, and 30 s at 71.7 °C for extension) in a final volume of 10 μL with 100 ng of cDNA, 200 nM primers, and 0.05 U/mL LA *Taq *DNA polymerase under the conditions recommended by the manufacturer (TaKaRa Biochemicals, Tokyo, Japan). PCR products were electrophoresed on 2.0% agarose gel and stained with ethidium bromide. The intensity of bands was quantified by the ImageJ software (National Institutes of Health, Bethesda, MD, USA) and data are expressed as the ratio of CYP2E1 mRNA to β-actin mRNA.

### 2.6.Statistical Analysis

Comparisons in the effect of resveratrol on hydroquinone-induced cytotoxicity and the expression of CYP 2E1 mRNA were performed using a one-way analysis of variance (one-way ANOVA) followed by the multiple comparisons, using a Windows version SPSS 15.0 statistical program package (SPSS Inc., Chicago, IL, USA).

## 3. Results and Discussion

### 3.1. Resveratrol Inhibited Hydroquinone-Induced Apoptosis in Mouse Primary Hepatocytes

**Figure 3:**
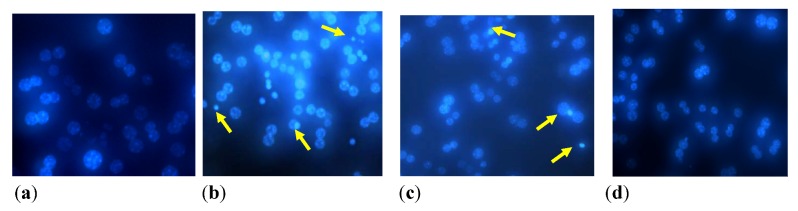
Fluorescence microscope images of hydroquinone-untreated (**a**) or treated (**b**) hepatocytes stained by Hoechst 33342. In comparison with the negative control (**a**), 48 h treatment of hydroquinone (**b**) caused morphological shrinking in hepatocytes, and apoptotic bodies and chromatin condensation were formed (indicated by arrows). Pretreatment by (**c**) 0.5 mM or (**d**) 1 mM of resveratrol 1 h before hydroquinone exposure inhibited hydroquinone-induced apoptosis.

Figure 3 shows that 48 h exposure to hydroquinone induced cell shrinkage, chromatin condensation, and nuclear fragmentation in mouse primary hepatocytes. Exposure to 100 µM hydroquinone caused 2.5 times of chromatin condensation in comparison with the control (data not shown). However, no dose-dependent response was observed. Hydroquinone induced apoptosis was markedly decreased in the cells by resveratrol pretreatment (1 h before hydroquinone exposure), particularly by 1 mM of resveratrol pretreatment.

Some researchers have found that hydroquinone caused apoptosis in human neutrophils, eosinophils, lymphocytes, HL-60, and Jurkat cells through both caspase-dependent and independent mechanisms [18,19,20]. Our findings were supported by these reports. Inayat-Hussain *et al*. indicated that both caspase-dependent and independent mechanisms should be considered in the intrinsic apoptotic pathway induced by hydroquinone [18]. Recently, Lee *et al.* reported that hydroquinone induced apoptosis in human by activating caspases 9/3 through caspase 9/3 pathway [20]. Zheng *et al*. found that resveratrol pretreatment significantly reduced H_2_O_2_ induced apoptosis in human lens epithelial cells [21]. Our study also proved that hydroquinone could cause apoptosis in mouse primary hepatocytes and pretreatment of resveratrol is capable of protect the cell against it.

### 3.2. Resveratrol Inhibited Hydroquinone-Induced Cytotoxicity

**Figure 4:**
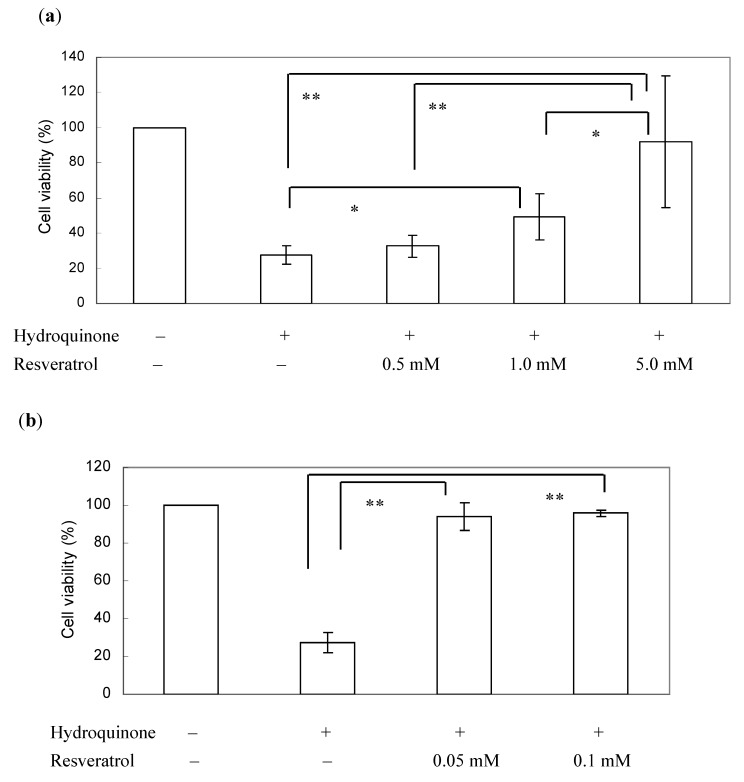
Effect of resveratrol on hydroquinone-induced hepatocyte toxicity. (**a**) Hydroquinone (0.4 mM) was exposed to the cells for 2 h at 37 °C. Resveratrol was added to the cells 1 h before hydroquinone exposure and was contained throughout the experiments. (**b**) Longer time of resveratrol pretreatment (24 h) at lower concentrations. Data are expressed as mean ± SD (n ≥ 6). Comparisons in effect of the resveratrol on hydroquinone-induced cytotoxicity among concentrations were performed using a one-way ANOVA followed by Tukey’s multiple comparisons. * *p *< 0.05, ** *p *< 0.01.

Figure 4 demonstrates that 400 µM hydroquinone exposure alone resulted in a significant drop of cell viability compared with the control. The addition of resveratrol to a final concentration of 0.5 mM 1 h before hydroquinone exposure did not show a significant improvement in survival rate of the hepatocytes, however, 1 mM or 5 mM of resveratrol treatment before hydroquinone exposure increased cell viability in a dose-dependent manner, and in particular the survival rate of the hepatocytes was recovered from 28% to near 100% by 5 mM resveratrol treatment. Interestingly, pretreatment with resveratrol for longer time (24 h) even in very low concentrations (50 μM, 100 μM), blocked the damage of hydroquinone to the cells.

It has been reported that hydroquinone-induced cytotoxicity was accompanied by ROS formation, particularly H_2_O_2_ formation [6,7,22]. An imbalance between cellular antioxidant defense capacity and ROS level could induce or suppress the expression of many genes [23]. Resveratrol is known to be capable of either scavenging free radicals directly or exhibiting antioxidant capacity indirectly through inducing antioxidant and detoxifying enzymes [11,21,24]. Zhang *et al*. found that resveratrol could activate the antioxidant transcription factor nuclear factor erythroid 2-related factor 2 (Nrf2) that involves the increased expression of many antioxidant and detoxifying genes like NAD(P)H:quinone reductase-1, and heme oxygenase-1 and the maintenance of the cellular redox homeostasis [25]. The work by Ungvari *et al*. demonstrated that resveratrol treatment prevent the endothelial cells against increase in intracellular H_2_O_2_ and H_2_O_2_-mediated apoptotic cell death induced by xenobiotics [26]. In the present study, pretreatment with either higher concentrations of resveratrol (≥1 mM) for 1 h or lower concentrations (50 μM, 100 μM) for longer time (24 h) markedly inhibited hydroquinone cytotoxicity and increased the cell viability. The former might imply a direct radical scavenging by resveratrol; the later possibly indicates an induction of antioxidant and detoxifying enzymes, which increases cellular oxidative stress resistance.

Several works also demonstrated that *in vivo* resveratrol could increase mitochondrial complexes in skeletal muscle, liver, and blood vessels [27,28,29]. It is known that the mitochondrion, the energy-converting organelle of eukaryotes, is often the target for toxic compounds [30]. Mitochondrial biogenesis is suggested to be involved in the regulation of cellular metabolism, redox regulation, and signal transduction [31]. The present study detected cell viability using WST-8 cell counting kit, based on the reduction of the formazan dye by the cellular dehydrogenase, in which NAD(H), NADP(H), and mitochondrial activity are also involved [16]. The increased survival by resveratrol pretreatment in the present study might also imply that resveratrol might protect the cells against hydroquinone-induced cytotoxicity through enhanced mitochondrial biogenesis. 

**Figure 5:**
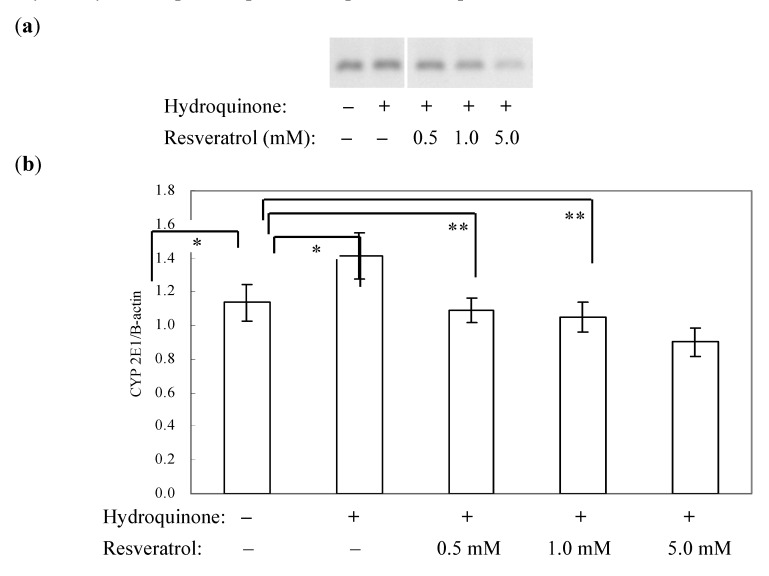
The effect of resveratrol on the expression of CYP2E1 mRNA in mouse primary hepatocytes exposed to hydroquinone. (**a**) Comparison of the expression of CYP2E1 mRNA among each treatment groups. (**b**) Comparison of the ratio of CYP2E1/β-actin mRNA among each treatment groups. Data were analyzed by one-way ANOVA followed by Tukey’s multiple comparisons. * *p *< 0.05, ** *p *< 0.01.

### 3.3. Resveratrol Suppressed Hydroquinone-Induced Expression of CYP2E1 mRNA

We further observed how resveratrol treatment could affect metabolic enzyme cytochrome P450 2E1 expression that is responsible for the metabolism of many oxidative xenobiotics [32]. We found that relative to untreated primary hepatocytes, 2 h hydroquinone exposure showed an increase in CYP2E1 mRNA level (Figure 5), whereas 1 h pretreatment of various concentrations of resveratrol before hydroquinone exposure to the cells suppressed the expression of CYP2E1 mRNA in dose-dependent manner (*p* for Trend: 0.001). In particular, the pretreatment of 5 mM resveratrol markedly downregulated hydroquinone-induced CYP2E1 mRNA level. 

Cytochrome P450 2E1 is one of the phase I xenobiotics metabolizing enzymes and it can generate ROS as byproducts after being bioactivated [33,34]. An elevated expression of CYP 2E1 mRNA in primary hepatocytes after hydroquinone exposure and a decreased expression of this enzyme when pretreated the cells with resveratrol in our study probably imply that resveratrol could effectively modulate hydroquinone-induced cytotoxicity through its antioxidant activity and suppressive effect on the expression of CYP 2E1. Our result of inhibitory effect of resveratrol on CYP 2E1 expression was consistent with the one by Piver *et al*. [35]. 

## 4. Conclusions

The present results demonstrated that resveratrol protected hepatocytes against hydroquinone toxicity and suppressed hydroquinone-induced expression of CYP 2E1 mRNA in a dose-dependent manner. These findings suggest that resveratrol protected the cells against hydroquinone-induced toxicity through its antioxidant function and possibly suppressive effect on the expression of CYP 2E1. 

However, concerning the suppressive effect of resveratrol on the expression of CYP 2E1, further experiments are needed to reach a conclusion.
